# Health services for reproductive tract infections among female migrant workers in industrial zones in Ha Noi, Viet Nam: an in-depth assessment

**DOI:** 10.1186/1742-4755-9-4

**Published:** 2012-02-27

**Authors:** Le Anh Thi Kim, Lien Thi Lan Pham, Lan Hoang Vu, Esther Schelling

**Affiliations:** 1 Department of Epidemiology and Biostatistics, The Hanoi School of Public Health, 138 Giang Vo Street, Ba Dinh District, Ha Noi, Viet Nam; 2 Swiss Tropical and Public Health Institute & University of Basel, Basel, Switzerland; 3 Long Bien District Health Center, 485B Ngo Gia Tu Street, Long Bien District, Ha Noi, Viet Nam; 4 Department of Epidemiology and Biostatistics, The Hanoi School of Public Health, 138 Giang Vo Street, Ba Dinh District, Ha Noi, Viet Nam; 5 Swiss Tropical and Public Health Institute & University of Basel, Socinstrasse 57, P.O. Box, 4002 Basel, Switzerland; 6 The Hanoi School of Public Health, 138 Giang Vo Str., Ba Dinh Dist., Ha Noi, Viet Nam

**Keywords:** RTIs, STIs, Female migrants, Industrial zones, Health care services, Viet Nam

## Abstract

**Background:**

Rural-to-urban migration involves a high proportion of females because job opportunities for female migrants have increased in urban industrial areas. Those who migrate may be healthier than those staying in the village and they may benefit from better health care services at destination, but the 'healthy' effect can be reversed at destination due to migration-related health risk factors. The study aimed to explore the need for health care services for reproductive tract infections (RTIs) among female migrants working in the Sai Dong industrial zone as well as their services utilization.

**Methods:**

The cross sectional study employed a mixed method approach. A cohort of 300 female migrants was interviewed to collect quantitative data. Two focus groups and 20 in-depth interviews were conducted to collect qualitative data. We have used frequency and cross-tabulation techniques to analyze the quantitative data and the qualitative data was used to triangulate and to provide more in-depth information.

**Results:**

The needs for health care services for RTI were high as 25% of participants had RTI syndromes. Only 21.6% of female migrants having RTI syndromes ever seek helps for health care services. Barriers preventing migrants to access services were traditional values, long working hours, lack of information, and high cost of services. Employers had limited interests in reproductive health of female migrants, and there was ineffective collaboration between the local health system and enterprises. These barriers were partly caused by lack of health promotion programs suitable for migrants. Most respondents needed more information on RTIs and preferred to receive these from their employers since they commonly work shifts - and spend most of their day time at work.

**Conclusion:**

While RTIs are a common health problem among female migrant workers in industrial zones, female migrants had many obstacles in accessing RTI care services. The findings from this study will help to design intervention models for RTI among this vulnerable group such as communication for behavioural impact of RTI health care, fostered collaboration between local health care services and employer enterprises, and on-site service (e.g. local or enterprise health clinics) strengthening.

## Background

According to United Nations Programme on HIV/AIDS (2001), migrants are people who move between places temporarily or permanently, by option or forced [1]. More roughly, migration can also be divided in domestic or international migration. In Viet Nam, after the introduction of the reforms started in 1986, the economic system switched from budget subsided to market-oriented economy, which enhanced investments and development. The transformation has led to a significant economic growth and poverty reduction. However, similar to other South East Asian countries, unbalanced development between rural and urban areas triggered migration flows from the countryside to cities [2]. For example Ha Noi, the second largest city in Viet Nam, has had a gradually increasing in-migration since 1986. Up to 2003, its population grew in average by 55.000 people each year of which 35-39% were migrants [3].

Rural-to-urban migration involves a high proportion of females because job opportunities for female migrants have increased in urban areas in the formal sector such as textile, footwear and garment factories, particularly in industrial zones [4-7]. Since 2009, the proportion of females is higher than that of males in most groups of migrants (e.g.. rural-to-urban and rural-to-rural) [7,8]. Average income of female migrants working in industrial zones has been much higher than the national poverty standard. However, after paying for room rent, electricity, water, and support to their families in rural areas, the remaining available budget for food and other fundamental living items is small. Still, female migrants' main difficulties are beyond "income" or "expenditure", but rather in "social integration" [9].

In 1979, Hull [10] reviewed the relationship between migration and health on a global level. The author coined the term of "healthy migrant syndrome", which implies that migrants seem to be healthier than non-migrants. Some authors also called this phenomena the "healthy migrant effect" [11,12] and was explained by: (i) migrants need to have a good health to undergo an arduous journey of migration and to comply with job requirements and working conditions at destination and (ii) migrants may move to benefit from better health care services at destination. However, Kristiansen et al. (2007) stated that the effect would fade out over time because migrants are exposed to many health risk factors at destination [13]. The report of World Health Organization (WHO) (2010) also identified that migrants are more susceptible and vulnerable to ill-health effects and have more limited access to health care (they seek care more rarely or cannot pay for them) [14].

In Viet Nam, Van Landingham (2003) indicated that rural-to-urban migrants need to cope with negative effects on almost all aspects of health such as physiology, psychological, emotional, physical, knowledge and conception about general health [15]. The 2004 Viet Nam Migration Survey led to the identification of migrant-associated health problems when compared to the general population, such as unhealthy status, less access to health care, lack of knowledge about reproductive health and sexually transmitted infections (STIs). For instance, the majority of female migrants aged 20-29 had no knowledge of reproductive health infections (RTIs), STIs, and HIV/AIDS [16].

RTIs include STIs, endogenous genital tract infections (eg. bacterial vaginosis and candida), and iatrogenic infections (eg. intrauterine device insertion). The latter is one of the leading causes of prenatal morbidity and mortality in developing countries [17]. RTIs can result in pelvic inflammatory diseases, infertility, adverse pregnancy outcomes, carcinoma and increased susceptibility to HIV [17]. Because of the severe morbidity of RTIs, early detection and treatment is important. However, it is difficult to distinguish between STI and non-STI clinical symptoms, also for health practitioners. Therefore, WHO recommends the management of symptomatic STIs/RTIs with a problem-based approach. The guideline may be modified to the context of a country [17]. Consequently, the Viet Nam Ministry of Health has adopted this guideline in 2010 in its National Guidelines for Reproductive Health Care Services [18], where STI/RTI syndromes consist now of vaginal discharge, lower abdominal pain, genital ulcers, genital warts, and inguinal lymph nodes.

WHO (2005) and the Viet Nam Ministry of Health (2010) also stated that education on STIs/RTIs and counseling play crucial roles for management of STIs/RTIs. Particularly, the Ministry of Health has identified the necessitiy of community education to improve RTI prevention and health service utilization through raising awareness about RTIs and changing perceived barriers to health care utilization [17,18].

In Viet Nam, the RTI intervention program was integrated in the family planning program. However, this program has rarely targeted female migrants; indeed, in contrary given their non- registration [19]. Although a number of previous studies showed that female migrants have faced many health problems including RTIs [16,20-22], the needs in information for and utilization of RTIs services of female migrants in reproductive age (i.e. aged 15-49 years old) has not yet been investigated. This paper attempts to address this gap and specifically focuses on female migrants working in industrial zones.

We present the results of two research objectives: (i) to examine the use of health care services for RTIs of female migrants; and (ii) to describe barriers related to the use of health care services. This project is embedded in a larger study on access to social services of inter-provincial migrants.

## Methods

### Study sites

The study was conducted in Long Bien district, Ha Noi, Viet Nam. The district is the gateway to Ha Noi, lies within the economic axis of Ha Noi - Hai Phong - Quang Ninh and involves many national highways. Moreover, many new urban compounds and industrial zones named Sai Dong A and Sai Dong B have been built in the district. All these have contributed to the dramatic economic development of Long Bien. Subsequently, it also attracted many inter-provincial migrants. The in-migration population in Long Bien was 22,143 persons in 2010, accounting for about 10% of the whole population.

There are 14 communes in the district and its health system was divided administratively into district and commune level. At district level, there are 4 public health centers (Duc Giang, Giao Thong Van Tai, Hang Khong, and Long Bien) and 5 private health centers. The public and private health centers are responsible for general health care services, including reproductive health. Each commune has a commune health center that is responsible for primary health care and national health programs, e.g. reproductive health and the expanded immunization program.

Study sites included the resident areas of two communes (Sai Dong and Thach Ban) in the district. These two study sites were chosen because they receive a substantial number of migrants working in the Sai Dong industrial zones.

### Study participants

The inclusion criteria of participants were: (1) a female migrant who moved from another province to Ha Noi, (ii) and was 18-49 years old, (iii) and continuously lived in a study site for 6 months-5 years, and (iv) worked in the Sai Dong industrial zones. The third criterion enables us to compare our results with other studies because most studies on migration in Viet Nam have recruited migrants moving to destination within 5 years prior to the studies. In addition, 6-month duration of migration also enables us to capture RTI illness of female migrants and their use of RTI health care.

### Study design and sampling

This study was a cross sectional design with mixed method approach combining both qualitative and qualitative methods. For the quantitative survey, a cohort of 300 female migrants was interviewed. These migrants were selected randomly from the sampling frame of all 1200 migrants currently located in the study area (the sampling frame was provided by the heads of the residential units, including both registered and non-registered migrants). We have calculated the sample size for a proportion and to estimate with an absolute precision, the sample size was calculated with the following parameters: anticipated prevalence of RTI as 25%, an absolute precision of 5% and an estimated non-respondent rate of 10%. Then the sampling interval was calculated and the first subject selected with a random number before each 4^th ^person on our sampling frame was contacted.

The qualitative survey consisted of 20 in-depth interviews with key informants and two focus group discussions (FGDs) with female migrants. The participants were recruited by targeted sampling. The interviewees were representatives of health centers (e.g. directors of and seniors in District Health Centers, Department of Health Information and Education, Department of Reproductive Health Care, Department of Public Health, Department of Disease Control and HIV/AIDS Prevention, and the heads of the commune health center), employers (e.g. director and health staffs of manufactories), local organizations (e.g. district and commune police, people committee, Population - Family Planning, Women Union, Labor-Invalids and Social Welfare, and Youth Union) and landlords of buildings with female migrants. Two FGDs included each 8 female migrants selected by the same inclusion criteria as in the survey.

We have explained to all study participants about the purposes of the study and they have signed a consent form. The study had also obtained the approval of the Institutional Review Board of Hanoi School of Public Health.

### Questionnaire and terminology

The in-depth interview catalogues, the FGD catalogues and the questionnaires were developed by using the ACCESS Framework [23] and tested in a pilot study. The 'Health Access Livelihood Framework' combines health service and health-seeking approaches and hence situates access to health care in the broader context of livelihood insecurity. Whether people actually recognize an illness and seek treatment depends to a large extent on their access to livelihood assets of the household, the community, and the wider society. Once they decide to initiate treatment, access becomes a critical issue. The degree of access reached depends on the interplay between i) the health care services and the broader policies, institutions, organizations, and processes that govern the services, and ii) the livelihood assets people can mobilize in particular vulnerability contexts [23].

The structured questionnaire with open and closed questions had five parts including: (i) background information such as age, ethnics, education, occupation, and marital status; (ii) migration information such as number of moves, migration reasons, and registration status and difficulties in registration; (iii) living conditions of participants such as house status, water supply, toilet, income and expenditure, working time, living difficulties and supports from their family, friends or local government; and (iv) their RTI symptoms, and (v) needs in and access to and utilization of information and health services.

The RTI symptoms were based on the syndrome approach described in The National Guidelines of Reproductive Health Care Services. These syndromes include abnormal vaginal discharge, genital wart, and genital ulcer syndrome [18]. The terminology of a "health care unit" and "the use of health care" were based on Viet Nam Health Care Law [24] and included both public and private services.

### Analysis methods

STATA version 10 was used to analyze quantitative data. Descriptive techniques such as frequencies and cross-tabulations were applied. We have used NViVo 7 to analyze the qualitative data. Findings from the qualitative part were used to triangulate and provide in-depth information to explain the results from the quantitative part.

## Results

The overall participation rate was high at 97%. Most female migrant respondents were young with a mean age of 24 years. The proportion of participants aged 30 or more was only 6.2% (Table 1). Table 1 also shows a high proportion of respondents with at least a high school education (84.6%) and about three-fourth respondents were single/unmarried, but 7.6% of these have ever had sexual relationship.

**Table 1:** Characteristics of female migrant workers (n = 291)

Categories	N	%	Categories	N	%
*Age*			*Duration of migration*		
18-24 years old	187	64.3	< 1 year	98	33.7
25-29 years old	86	29.5	1-3 year	109	37.5
≥ 30 years old	18	6.2	> 3-5 year	84	28.8

*Education*			*Temporal registration*		
Less than high school	45	15.5	Yes	249	85.6
High school	185	63.6	No	42	14.4
Higher than high school	61	21.0			

*Ethnicity*			*Health insurance*		
Kinh	278	95.5	Yes	229	78.7
Others	13	4.5	No	62	21.3

*Marital status*			*Source of water supply*		
Married	71	24.4	Tap water	216	74.2
Unmarried	220	75.6	Drilled-well water	75	25.8

*Sexual intercourse*			*Type of toilet*		
Yes	93	32.0	Private toilet	217	74.6
No	198	68.0	Sharing toilet	74	25.4

In the study, we found that 85.6% of the respondents were temporary registered and 78.7% had a health care insurance. In which, about one fourth of participants still use drilled-well water and sharing toilets (Table 1). Although the living conditions of female migrant respondents were seemingly poor, most of them felt satisfied with their living conditions. They preferred to make and save as much money as possible because their income is not only for themselves, but also for their family at the original areas.

*"... they rent a room, a small room. As you know, migrants often have low income. Therefore they usually share a room with others. Living condition is poor, of course. In fact, they take each day as it comes, they usually want to save money as much as possible. So they cannot have good living conditions" *(an officer of Labor - Invalids and Social Welfare).

*"We can live anywhere, it doesn't matter. You see, our salary is low, therefore we must pay for living as less as possible, we must save money" *(a 24-year-old female migrant, 3 years of migration).

A migrant should register with the local police at destination. Temporary registration - *dang ky tam tru - *at the destination is not complicated. For migrants who live at destination 3 months and longer, the local government requires a copy of the identity card and a registration form of migrants. Most female migrants were assisted for this by their landlords. This may explain the high proportion of registered respondents. However, female migrants often register only if they feel satisfied with their settlement and job. In other words, they casually switch their accommodation if they feel any inconvenience; for instance, they fail to liaise with the local community; or their rents are going up, or their work is too tough. These flexibilities make resident management of local government difficult.

*"I did the registration. Actually, my landlord did it. We gave him a copy of our identity cards" *(a 20-year-old female migrant, 1 year of migration).

*"I have not registered yet, because I want to find another room with a cheaper rent than my current room" *(a 19-year-old female migrant, 1 year of migration).

*"We gave them a sample profile for registration. They just gave us a copy of their identity card and their photo. That is it! Then we gave them their temporary registration card and a receipt of 10.000 VND *[about 0.5 US dollar]*. If they stay less than 3 months, they just need to confirm their stay with us, but do not need a registration card" *(a local policeman).

*"Because they *[female migrants] *do not consider to settle permanently, they change their accommodation frequently, due to for example conflicts with the landlords, with neighbors, with other migrants, or a higher renting cost. So you see, with any reason, they can switch and have unstable accommodations" *(a local manager).

The Viet Nam Law of Health Insurance stated that all employees having a labor contract with their manufactory must be covered by "obligatory health insurance". The law defines that employers must pay two-third and employees one-third of monthly health insurance fee. The fee does not exceed 6% of employees' monthly salary. The regulation contributes to ensure the high coverage of health insurance for female migrants working industrial zones (Table 1). However, some manufactories do not comply with the law, and not all female migrants understand their rights of having health insurance.

*"About 70-80% *[of enterprises issuing health insurances for their employees] *is quite good, I think. And migrants also do not understand their right. Or they do not care about it because they think they are still healthy and they do not need it" *(Manager, District Health Center).

*"Our company has contracts with foreign companies. So we should follow all regulations that ensure employers' right such as labor contracts, social and health insurance. ... If we do not do all these things, foreign companies would not contract with us. But you know, some companies such as small private companies do not care about this" *(a health staff of a State-owned manufactory).

*"I do not know, my manufactory gives me nothing *[health insurance]*" *(a 23-year-old female migrant, 1 year of migration).

*"I do not know that I can register my health insurance at commune health center here. My manufactory seems to have a contract with that center *[Duc Giang health center]*, and I think we should not change..." *(a 24-year-old female migrant, 2 years of migration).

*"All manufactories - small or large - should provide health insurance for their employees. The fee is not much. But employees usually do not know their rights, and they also do not dare to ask for the rights." *(an officer of the Labor - Invalids and Social Welfare).

### RTI symptoms and obstacles for access to RTI care services of female migrants

**Figure 1:**
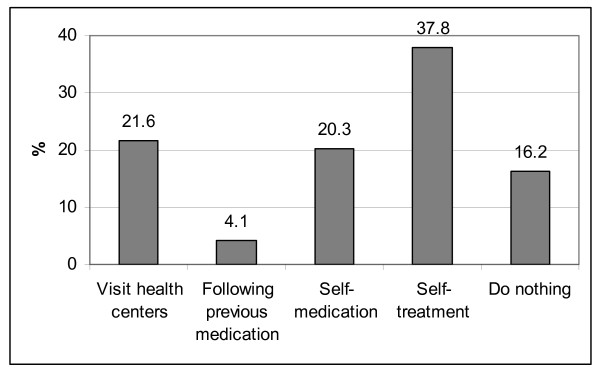
**Health-seeking practice among female migrant workers having RTI symptoms in the previous 6 months (n = 74, 25.4% of all female migrant respondents)**.

The reporting of one or several RTI symptoms (e.g. abnormal vaginal discharge, vaginal itching, and genital wart/ulcer) in the previous 6 months was recorded in 25.4% of interviews. However, only 16 respondents (21.6%) of those sought health care at a health center; the others did self-treatment (i.e. washing their genital area with feminine hygiene fluid) (37.8%), or self-medication (20.3%), or did nothing (16.2%) (Figure 1).

### Traditional health beliefs and values: unmarried women seem to have no risk of RTIs

The perception for RTIs of female migrants seemingly partly explains this health seeking pattern. Most respondents with symptoms thought these symptoms to be normal and not a sign of a disease. They also often stated that they can bear these symptoms, do not need to seek for health care, or they can treat themselves by using feminine hygiene fluid. This seems to indicate that female migrants do not have comprehensive knowledge about RTIs, its severity and subsequent health problems.

*"I am ok, why do I have to go to a doctor? When it was too itchy, I went to a drugstore and bought a bottle of feminine hygiene fluid, it will be fine after few days of using the fluid. So do all of my friends" *(a 20-year-old female migrant, 2 years of migration).

*"It is *[the symptoms] *not too serious to seek for health care ... (laughing) ... We are totally healthy, strong like a cow or a buffalo (laughing)" *(a 25-year-old female migrant, 2.5 years of migration).

Female migrants thought that gynecological examination was only for married women, not for unmarried ones. Single female migrants stated that they were free of risk for any gynecological disease. Female migrants also perceived an unmarried woman - expected by the society to be a virgin - with an RTI to be "bad". In other words, they commonly thought that RTIs are a consequence of sexual intercourse. As a result, they feel quite embarrassed as getting a gynecological examination.

*"It is *[gynecological examination] *only for married women; we are unmarried so..." *(a 22-year-old female migrant, 2 years of migration).

*"I am not married yet, so I am too ashamed to have a gynecological examination. If my friends knew... they would think I am promiscuous" *(a 18-year-old female migrant, under 1 year of migration).

*"There is no need to visit health facilities ... they are only for married women, not for unmarried girls like us, so we don't go there ...*" (a 22-year-old female migrant, 2 years of migration).

### Availability, accessibility, and affordability of RTI care services and lack of information about health care insurance

In addition to the thinking of RTIs being normal or gynecological examinations only to be for married women, for female migrants there are other reasons to not go to health centers, despite the fact that a high proportion had a health insurance. In fact, there are different health care services that female migrants can use. Public health system includes manufactory health centers, commune health centers, district health centers/hospitals, and health centers where they have their insurance registration (mostly district health centers). The private health system consists of private clinics, private health centers or hospitals. However, most female migrants do not go to private health centers because of the higher costs. Meanwhile, health centers where female migrants register their health insurance are often far from their home. The health insurance is registered at a certain district health center that it has a health care contract with the manufactory. Employees have the right to register their insurance at another health center, also in another administrative unit. But they do not know about this.

*"I live here, but they *[the manufactory] *registered my health insurance at Duc Giang hospital *[a district hospital]*. It is quite far. If I go there to get examination, it will take half of day. Thus I can't, I need to work" *(a 24-year-old female migrant, 3.5 years of migration).

*"Alike other residents living here, they could register their health insurance here to get medical examination. If they do it, they will have all health services under health insurance regulations. If they don't, they will pay all health care costs" *(a health staff of a commune health center).

*"I don't know. Where they *[the manufactory] *registered my health insurance, where I must go to have medical examination. Could I change? Yes? I didn't know this" *(a 20-year-old female migrant, 1.5 years of migration).

### Adequacy of RTI care services

Moreover, health centers usually provide general medical examinations during the working time. Therefore, if female migrants want a genital examination at a health center, they need to take out half or one of their working day. However, this means that a half-day salary is lost, which is another barrier to health care.

*"We work in the third shift. We come back home in the morning and sleep until noon. In the afternoon, we eat and sleep again. And then go to work. We have no time for medical examination" *(a 22-year-old female migrant, 2.5 years of migration).

*"We work in shifts. We could ask the manager to get absence time for medical examination, but we are afraid.... And when we finish our work at the manufactory, hospitals are closed" *(a 20-year-old female migrant, 2 years of migration).

"As you know, they just want to work. They don't want to be absent from their work, they are afraid of being excluded from the wages, from bonuses... They are mostly poor, thus we treat them for the love, not for money. We sometimes give them drugs from national health programs for free. Or we just charge for clinical examination. It is not much. If they have children under six years old*, we treat their children for free. But the important thing is that they should care for their health" (a health staff of commune health center).

*: In Viet Nam, every child under six years old has an insurance card for all treatments for free of charge.

### Common health care of employers

In fact, many employers usually provide to their employees annual medical examination; also because this is required by the Labour Law; however, the examinations are often a more formal matter. Reproductive tract diseases such as RTIs/STIs are commonly ignored, especially for unmarried female migrants.

*"Doctors *[doing the regular examination at work] *just have a quick look at a person to find something abnormal on the surface or anything else. They do not check it *[genital area] *and do not ask about it*..." (a 26-year-old female migrant, 3 years of migration).

*"We focus mainly on labour safety and prevention of occupational diseases, such as occupational deafness and respiratory problems... We do not care much about reproductive health of women. We have given them health insurance cards, so if they have any health problem, they have to seek for treatment at health facilities. If they have some benign symptoms, we give them some medicine, that's all..." *(Health staff of a foreign company).

The collaboration between the local public health services and manufactories in providing information and health care for RTIs to female migration was limited, despite the fact that the district health center held meetings with the manufacturers in the industrial zone every 3 months. These meetings would be an opportunity for both the local health system and manufactures to harmonise their approaches to address health problems, but the meetings have not attracted much attention of most companies, particularly of the foreign ones.

*"We have already integrated communication and provided condom for free at some enterprises, however, they are only State-owned enterprises, but not foreign enterprises. We have not accessed any international enterprise*" (a representative of Department of Disease Control and HIV/AIDS Prevention).

*"We used to contact the enterprises to organise communication sessions about reproductive health, but we have failed. It is too difficult to do. Their employees work in shifts, so they cannot spend time for these things. We do want to do this, but we cannot, it's so hard" *(a representative of Department of Reproductive Health).

*"Only *[a State-owned enterprises] *came often *[to the meeting]*. Others did not. We could not manage them. We are a professional agency, not an administrative agency, we have no legitimacy to manage them. We have no right to ask them that they come to regular meetings. No. They should be active" *(a representative of Department of Public Health).

### Needs and utilization of RTI information and health service

However, female migrants thought that seeking health care services is necessary, particularly when they have several symptoms. Table 2 indicates that most female migrants (75.6%) expect to get gynecological examination at health centers. However, 63.2% of respondents need a gynecological examination that is fully subsidized, 31.3% need some subsidization, and only 5.5% need none (Table 2). On the one hand, they want to save their expenditure; on the other hand, and they want to be "safe" or "unembarrassed". The latter reason is explained by respondents that if RTI care services are free of charge or partly subsidized, many females would seek public health care. Is so, they would feel less ashamed and more comfortable because they obviously would belong to a group with the same health problems.

**Table 2:** By female migrant workers used and sought reproductive health programs

Contents	N	%
**Sought health care services**		

*Location of services*	*291*	
At commune level	220	75.6
No (other places)	71	24.4

*Subsidized*	*220*	
Totally subsidized	139	63.2
Partly subsidized	69	31.3
Not subsidized	12	5.5

**Needs on information**		

*Needs on reproductive health information*	*291*	
Yes	234	80.4
No	57	19.6

*Source of information*	*234*	
Television/radio	125	53.4
Peer/relatives	84	35.9
Book/newspaper/magazine/leaflet	104	44.4
Manufactory health officer	97	41.4
Local health worker	119	50.9
Loudspeakers	*68*	29.1
Others	*15*	6.4

*Sought place of communication sessions*	*234*	
At commune	91	38.9
At manufactory	193	82.5
No needs	26	11.1

**Local communication programs**		

*Local communication programs*	*291*	
Known	28	9.6
Unknown	263	90.4

*Participated in the program*	28*	
Yes	4	14.3
No	24	85.7

* only for female migrants who have known about local communication programs

*"I want to have gynecological examination, but I am not married yet, so I am embarrassed..." *(a 20-year-old female migrant, 2 years of migrant).

*"I want to visit health facilities; it is good to have gynecological examination because I do want to know what is happening, sometime it's *[her genital area] *not fine. If many persons such as my friends, my colleagues came there to have gynecological examinations, I would be totally fine, not embarrassed. But if not, no, it is so ashaming" *(a 20-year-old female migrant, 2 years of migrant).

*"We sometimes have genital examination programs free of charge for females at reproductive age. They come a lot. Yes, the programs are for both resident and migrant females. In fact, the programs are offered to registered persons, but they are all poor, we cover all. We just estimate a bit more needed resources (smiling)" *(a representative of Department of Reproductive Health).

In Viet Nam, health information campaigns including RTIs have been expanded to all "resident groups" via community health workers, resident group meetings and information distributed via loudspeakers. However, female migrants usually work shifts - and spend most of their day time at work. Thus they are more rarely reached by these information campaigns than the general population.

*"There is no loudspeaker in this street. There is one on the main road, but it is very far from here. I have not heard anything from it, because it's only broadcasted at 6 am, and at this time, we are still in the manufactories ..." *(a 27-year-old female migrant, 4 years of migration).

*"There may be some communication meetings integrated into resident group meetings, but no one invites us. But my work is also too busy, so I don't participate in any activity of resident groups, I am only at the manufactory*" (a 21-year-old female migrant, 3 years of migration).

*"They *[female migrants] *just focus on their work for earning more money. Therefore they don't participate into any information programs. We have invited them, but they don't join us. For example, we held some information sessions here *[at their *to dan pho - *"resident group"]*, but they did not come. The problem is that we want to help them, but they don't care. Only if they get very seriously sick, we will come" *(health staff of commune health center).

**Figure 2:**
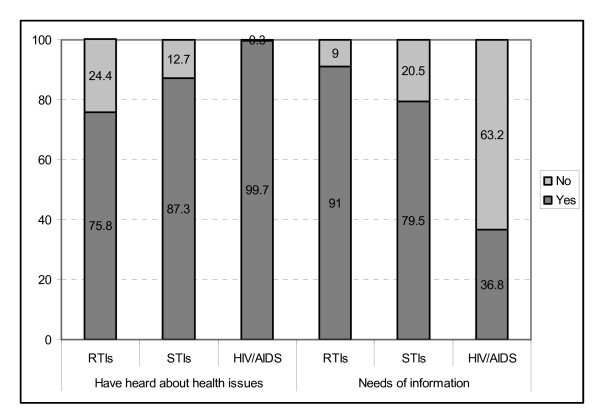
**Needs of information about RTIs of female migrant workers (n = 291)**.

Consequently, only 9.6% respondents knew about local information programs; in which only 4 women have ever participated. In order to improve perception of and information campaigns for RTIs, their needs were recorded. Most of respondents (80.4%) need information on reproductive health in general and on RTIs in particular (Table 2). Indeed, Figure 2 also shows that female migrants need more information about RTIs and STIs than on HIV/AIDS. Also, 82.5% of female migrants wanted to receive more information from their employers. Disseminating information at the home domiciles of migrants, if possible, should be on Saturdays or Sundays, mainly in the afternoon or evening when migrants could be at home.

*"In general, it's better to provide information at manufactory because we work there, we have more time and it is easier for us to access information. If they do it here *[at the health commune]*, it should be on Saturdays or Sundays" *(a 26-year-old, 3 years of migration).

## Discussion

The current study attempted to capture the needs and utilization of health care service for RTI of female migrants at reproductive age working in an industrial zone in Viet Nam. In this study, we found that female migrants working at industrial zones are young. Moreover, they are younger than female seasonal labours and have higher education levels than female seasonal labours [21]. In fact, migrants working in industrial zones (inter-provincial migrants) have a higher than needed education level for labour requirements of enterprises while most seasonal labours are rural-to-urban migrants who seek a job during harvest shifting time and thus they are mainly married and over 30 years old [21]. We also found a high proportion of female migrants having health insurance. The reason for this is that most employers should provide health insurance to employees by law [24].

The proportion of unmarried female migrants, who have had sexual intercourse in the study (7.6%). We have used female interviewees who could comfort the women that all data will be treated confidentially, but it is important to note that our proportion of sexual partnerships might be higher because having sexual intercourse for unmarried females was a sensitive issue and thus they usually tended to not respond to the question.

There was up to date no report about prevalence of RTIs or RTI symptoms for female migrants in Viet Nam. We have found that a quarter of the 291 respondents reported RTI symptoms. However, this proportion is lower than the proportion of other studies among settled communities in the Mai Dich, Ha Noi (33.7%), a study at Hoai Duc, Ha Tay (58.3%), and a study at Quang Tri (26.4%) [25-27]. Unlike the current study with mainly young single migrant women, participants of other studies were married non-migrant women. In these studies, clinical examination depicted a higher proportion of RTIs when compared to reported symptoms; for instance: 62.1% in Mai Dich, 53.1% in Hoai Duc, and 63.8% in Quang Tri. Difference between clinical detection rates than reported rates of RTIs could be caused by the fact that many RTIs often have calm symptoms or no felt symptom. Thus, the true prevalence of RTIs of female migrants in the study could be higher.

Among female migrants with RTI symptoms, the proportion of visiting a health center in our study was rather low (21.6%). This proportion is much lower than studies among non-migrant women in Hoai Duc, Ha Tay, and some Northern provinces, 48.2% and 48.6% respectively [25,26]. In contrast, the proportion of self-medication and self-treatment in the current study was higher than in the two latter studies. Note that in contrast to these studies most of our female migrants were unmarried, and they believed that RTIs is a health problem of married women alone.

Indeed, Ngo et al. (2007) and Lan et al. (2008) showed that most Vietnamese women go to drugstores/pharmacies to seek medication against their RTIs/STIs or some advice from drug sellers before going to health care centre [28,29]. Drugstores and pharmacies are less expensive than the health centres (in the case of without health insurance). In addition, given their larger number, access is easier and women also feel less ashamed. However, several studies indicated that not all treatments for RTIs/STIs at the pharmacy are in compliance with national guidelines for RTIs/STDs treatments [30].

The World Health Organization (WHO) recommended to reduce self-medication or self-treatment via buying drugs from pharmacies without a doctors' consultations [31]. The main reason is that drug sellers are not trained for medical treatment and thus they are not able to sell drugs effectively or correct dose. Although symptoms may (temporarily) disappear after such a treatment, the infections or diseases may not yet be cured. As a consequence, it also makes bacteria become resistant to antibiotics because they do not follow-up on their clients as a doctor would do on his patients.

One major barrier for female migrants to seek RTI health care was their embarrassment. This confirms other recent studies showing that stigma, shamefulness, and embarrassment are factors preventing people with RTI symptoms from seeking for treatment at health facilities [32-34]. Therefore, female migrants wanted to use RTI health care services in local community, but they also wanted to have clinical examination without fee or with some subsidization. This would attract more women to go to a health facility without them feeling ashamed.

Similarly to other recent studies on female migrants, most female migrants had heard of RTIs, STIs, and HIV/AIDS from a variety of information sources, but mainly from mass media [22,35]. We could show some limitations of local communication programs and health care activities for employees of enterprises. Female migrants usually work in shifts, thus they can hardly participate in communication events at the community level which would be a rich source of information. Therefore, female migrants would prefer to have communication sessions at their manufactories or on week-ends.

Meanwhile, most female migrants wished to have more information about RTIs, rather than STIs, and HIV/AIDS. This is because they have received some information about the two latter health issues from communication campaigns that are ongoing since many years. In parallel, employers did not pay enough attention on reproductive health for female migrants. Moreover, the local health system has not effectively collaborated with health care activities of manufactories. All these factors lead to central barriers to seek information and health care services to female migrants.

It is important to note that the study collected information of RTI symptoms by a questionnaire but not clinical examination. In fact, RTIs are still quite sensitive for female migrants, especially unmarried females. Thus, clinical checks in this study seem to be unfeasible. In addition, study conducted in Sai Dong industrial zone only. The zone is similar to other industrial zones in Viet Nam, but it is difficult to generalized about need for health care services for RTIs among female migrant workers and their services utilization in the whole country.

## Conclusion

The current study indicated that the use of RTI health care services among young female migrant workers was limited. It also identified barriers to health care seeking behaviour of female migrants. The barriers included both perspective of users and potential best providers: too little awareness and understanding of female migrant workers needs regarding RTIs and RTIs treatment, limited interests of employers in reproductive health of female migrants, and ineffective collaboration between the local health system and enterprises.

The study also indicated crucial needs of access to information and services - and their utilization - for RTIs of female migrants. Communication for behavioural impact of RTI health care should be fostered and the communication campaigns need to be conducted at manufactories where female migrants spend most of their time. In addition, it is necessary to improve collaboration between local health system (i.e. district health center) and manufactories in the same administrative units.

## Abbreviations

FGDs: Focus group iscussions; RTIs: Reproductive health infections; SAVY: Survey Assessment of Vietnamese Youth; STIs: Sexual transmission infections; WHO: World Health Organization.

## Competing interests

The authors declare that they have no competing interests.

## Authors' contributions

ATKL carried out the study design, data analysis, drafted and completed the manuscript. LTLP carried out the study proposal, participated in data collection, and carried out data analysis. LHV participated in the data analysis and reviewed the manuscript. ES conceived the study, and participated in its design and reviewed the manuscript. All authors have read and approved the final manuscript.
